# *Wolbachia* Infection Alters the Microbiota of the Invasive Leaf-Miner *Liriomyza huidobrensis* (Diptera: Agromyzidae)

**DOI:** 10.3390/microorganisms13020302

**Published:** 2025-01-30

**Authors:** Ya-Xin Duan, Ying-Hua Zhuang, Yu-Xin Wu, Tian-Wei Huang, Zhang-Rong Song, Yu-Zhou Du, Yu-Xi Zhu

**Affiliations:** 1 Department of Entomology, College of Plant Protection, Yangzhou University, Yangzhou 225009, China; 221804305@stu.yzu.edu.cn (Y.-X.D.); 221804327@stu.yzu.edu.cn (Y.-H.Z.); 221804318@stu.yzu.edu.cn (Y.-X.W.); 221804308@stu.yzu.edu.cn (T.-W.H.); 2 Entomology and Nematology Department, University of Florida, Gainesville, FL 32611, USA; zhangron.song@ufl.edu

**Keywords:** endosymbiont, *Wolbachia*, invasive pest, microbiome

## Abstract

Microbe–microbe interactions within a host drive shifts in the host’s microbiota composition, profoundly influencing host physiology, ecology, and evolution. Among these microbes, the maternally inherited endosymbiont *Wolbachia* is widespread in the invasive pest *Liriomyza huidorbrensis* (Diptera: Agromyzidae). However, its influence on the host microbiota remains largely unexplored. In the study presented herein, we investigated the bacterial communities of *Wolbachia* wLhui-infected (wLhui+) and -uninfected lines (wLhui−) of *L. huidorbrensis* using 16S rRNA gene high-throughput sequencing. For both leaf-miner lines, Bacteroidota was the dominant phylum (relative abundance: 59.18%), followed by Pseudomonadota (36.63%), Actinomycetota (2.42%), and Bacillota (0.93%). We found no significant differences in alpha-diversity indices between the wLhui+ and wLhui− lines (*p* > 0.05). However, principal coordinates analysis revealed significant differences in microbiota composition between the wLhui+ and wLhui− lines (PERMANOVA: *p* < 0.001), explaining 76.70% of the variance in microbiota composition. Correlation network analysis identified robust negative and positive associations between *Wolbachia* and several genera, suggesting that *Wolbachia* shapes microbial composition through competitive or cooperative interactions with specific taxa. Overall, our study suggests that *Wolbachia* plays a key role in shaping the leaf-miner microbiome, potentially affecting host fitness.

## 1. Introduction

Insects often harbor a variety of microbes that can influence key host traits, exerting either beneficial or harmful effects on their hosts [1,2,3]. The microbial communities associated with insects are typically highly diverse and variable [4]. Understanding the underlying factors shaping the insect microbiota is therefore essential for advancing insect microbial ecology and developing effective pest control strategies [5,6,7].

Both deterministic and stochastic factors contribute to the assembly of microbiota in many insect species [8,9,10,11]. Several studies have identified key deterministic drivers of the bacterial communities in insects, including host characteristics such as species [12,13], developmental stage [14], sex [15], and genetic background [16], as well as environmental factors like diet [14,17], temperature [18,19], and habitat [20]. All of these factors significantly influence the host microbiota. Emerging evidence also highlights the importance of microbe–microbe interactions in determining microbiota flexibility [4,21,22], where resident or intrinsic species can shape the overall diversity of the microbial community [21]. For example, the endosymbionts *Cardinium* affects microbial diversity and changes host phenotypes in the planthopper *Nilaparvata lugens* [23,24] and the whitefly *Bemisia tabaci* [25]. Similarly, *Wolbachia* and *Spiroplasma* have been shown to influence the bacterial community in the spider mite *Tetranychus truncatus* [26,27].

*Wolbachia* is a widespread, maternally inherited, facultative endosymbionts found in a wide range of insect and mite species [28,29,30]. It plays multiple roles in manipulating host reproduction, fitness, and other traits [7,31,32,33,34,35,36]. Previous studies have demonstrated that *Wolbachia* can influence the diversity and composition of bacterial communities in various insects, including planthoppers [37,38], mosquitoes [39], fruit flies [40,41], cabbage root flies [42], isopods [43], and spider mites [26,27]. However, some comparative studies have suggested that certain *Wolbachia* strains have no significant effect on the diversity or composition of the microbiota in mosquitoes [44] and flies [45]. One possible explanation is that the subtle effects of certain *Wolbachia* strains on the host microbiota may be due to these strains exhibiting reduced competition for resources and space with other microbes in the host [44,45]. Overall, these discrepancies across studies may arise from differences in host species or *Wolbachia* strains.

The leaf-miner *Liriomyza huidobrensis* (Blanchard) is a polyphagous invasive pest that threatens a wide range of vegetable and flower crops, posing significant risks to agricultural and natural ecosystems worldwide [46,47,48]. The microbiota of invasive species can facilitate host adaptation and expansion in various ways [49,50,51]. Concomitantly, their microbiome is also influenced by the novel environmental factors encountered during these invasions [52]. *Wolbachia* is prevalent in leaf-miners and has potential as a biological control agent [52,53,54]. Our research on the microbiota of *L. huidobrensis* has shown that the endosymbiont *Wolbachia* is the dominant bacterial taxon within the microbiota [52,55], and the assembly of this microbiota is strongly influenced by environmental factors [52,55]. However, there is still limited understanding of how the heritable endosymbiont *Wolbachia* affects the host microbiota.

In this work, we surveyed the bacterial community of *L. huidobrensis* infected with *Wolbachia* wLhui (wLhui+) and antibiotic-cured (wLhui−) individuals using high-throughput 16S rRNA amplicon sequencing. Our aim was to assess the influence of *Wolbachia* on the diversity and structure of the bacterial communities in *L. huidobrensis*. Ultimately, these findings provide valuable insights into the internal drivers of the host microbiota and offer promising avenues for improving invasive species management strategies.

## 2. Materials and Methods

### 2.1. Leaf-Miner Lines

The wild-type *L. huidobrensis*, naturally infected with *Wolbachia*, was originally collected in March 2023 from cowpea plants in Yunnan, China [55]. Upon arrival at the laboratory, we first established an isofemale line and maintained it on organic red bean (*Phaseolus vulgaris*) seedlings at the 2 expanded leaf stage. From the offspring of this isofemale line, 20 individuals were randomly selected to establish an *L. huidobrensis* line with 100% prevalence of the *Wolbachia* strain wLhui (denoted wLhui+). Another set of 20 individuals from the same generation was reared on bean seedlings soaked in tetracycline solution (1 mg/mL) for three generations to completely eliminate *Wolbachia* strain wLhui. This *Wolbachia*-free line (named wLhui−) was then reared without tetracycline treatment for more than 10 generations to allow for recovery and reduce the potential effects of tetracycline on the host microbiota. The *Wolbachia* infection status of each line was confirmed by qPCR, as previously described [56]. Both wLhui+ and wLhui− lines were maintained on organic red bean seedlings under the same laboratory conditions (25 ± 1 °C, 60% relative humidity, 16 h light: 8 h dark cycle). Newly emerged adult females and males from both lines were starved for 2 h to ensure that any food in their guts was egested, and then they were frozen at −80 °C in September 2024. Prior to experimentation, the *Wolbachia* infection status of the newly emerged adult individual was screened by PCR (Figure S1). Each individual sample was cleaned with 75% ethanol followed by sterile water before DNA extraction.

### 2.2. DNA Extraction and PCR Amplification

The total DNA was extracted from a single individual in October 2024 using the QIAGEN DNeasy Kit (Hilden, Germany), following the manufacturer’s specifications. The bacterial 16S rRNA V3-V4 region was amplified by PCR using the primers 341F (5′-CCTAYGGGRBGCASCAG-3′) and 806R (5′-GGACTACNNGGGTATCTAAT-3′) [11]. The PCR conditions were as follows: initial denaturation at 95 °C for 2 min, followed by 25 cycles of denaturation at 95 °C for 30 s, annealing at 55 °C for 30 s, extension at 72 °C for 30 s, and a final extension at 72 °C for 5 min. PCR reactions were performed in triplicate in 20 µL mixture containing 4 μL of 5 × FastPfu Buffer, 2 μL of 2.5 mM dNTPs, 0.8 μL of each primer (5 μM), 0.4 μL of FastPfu Polymerase, and 10 ng of template DNA. Amplicons were extracted from 2% agarose gels and purified using the AxyPrep DNA Gel Extraction Kit (Axygen Biosciences, Union City, CA, USA), following the manufacturer’s instructions.

### 2.3. Library Construction and Sequencing

Purified PCR products were pooled in equimolar concentrations before sequencing analysis. Sequencing libraries were constructed using the TruSeq DNA PCR-Free Library Preparation Kit, and paired-end sequencing (2 × 250) was performed on an Illumina MiSeq platform (Shanghai BIOZERON Co., Ltd., Shanghai, China), following standard protocols.

### 2.4. Bioinformatic Processing

Paired-end reads were merged and quality-filtered using QIIME 2 with default settings as described previously [11]. Quality-filtered reads were denoised and clustered into amplicon sequence variants (ASVs) using CD_HIT v4.5.7. After quality filtering and chimera removal, a total of 628,143 raw reads were obtained from 18 samples, with each sample containing between 29,713 and 38,001 reads. Taxonomic assignment of bacteria was performed using the uclust algorithm v1.2.22 against the SILVA database (Release138.2). Rarefaction curves indicated near-saturation of community coverage (good coverage > 99% for all samples; Table S1). To analyze alpha-diversity, the samples were rarefied to the same normalized sequencing depth (19,381 sequences) to ensure a random subset of ASVs for all samples.

### 2.5. Microbial Community Analysis

Statistical analyses and data visualizations were performed using MicrobiomeAnalyst 2.0 (https://www.microbiomeanalyst.ca/, accessed on 17 December 2024) [57] or GraphPad Prism version 10.00.

To compare significant differences in each alpha diversity index (e.g., observed ASVs, Shannon, Chao1, ACE, Simpson, and Fisher) between the wLhui+ and wLhui− lines, we employed *Mann–Whitney U* tests.

To assess variation in overall bacterial community composition between wLhui+ and wLhui− lines, we conducted a principal coordinates analysis (PCoA) based on a Bray–Curtis dissimilarity matrix. A permutational multivariate analysis of variance (PERMANOVA) was performed using 999 permutations to assess significant differences in bacterial community composition between the two lines.

For comparison of the bacterial composition between lines, the average relative abundance of the nine most abundant genera (average relative abundance > 1%) in individuals was determined and visualized in bar plots. Additionally, genera present in at least 50% of the samples within each line were considered as core genera. Significant differences in the abundance of specific microbes at the genus level between the wLhui+ and wLhui− lines were assessed using DESeq2, heat tree analysis, and linear discriminant analysis effect size (LEfSe) with default parameters in MicrobiomeAnalyst 2.0.

Finally, to investigate relationships between the relative abundance of bacterial genera, we performed correlation analysis. A correlation was considered statistically significant when *p* < 0.05, and the Spearman correlation coefficient (*ρ*) was >0.6 or *ρ* < −0.6.

## 3. Results

### 3.1. Overview of Sequencing Results

A total of 348,858 high-quality bacterial clean reads were obtained from 18 samples (9 wLhui+ and 9 wLhui−) of leaf-miners. After assembling and quality filtering, the dataset comprised 631 amplicon sequence variants (ASVs), which were classified into 6 phyla, 7 classes, 16 orders, 26 families, and 38 genera. In terms of relative abundance, Bacteroidota was the dominant phylum (59.18%), followed by Pseudomonadota (36.63%), Actinomycetota (2.42%), and Bacillota (0.93%) (Figure S2).

### 3.2. Effect of Wolbachia Infection on Microbial Community Diversity

**Figure 1:**
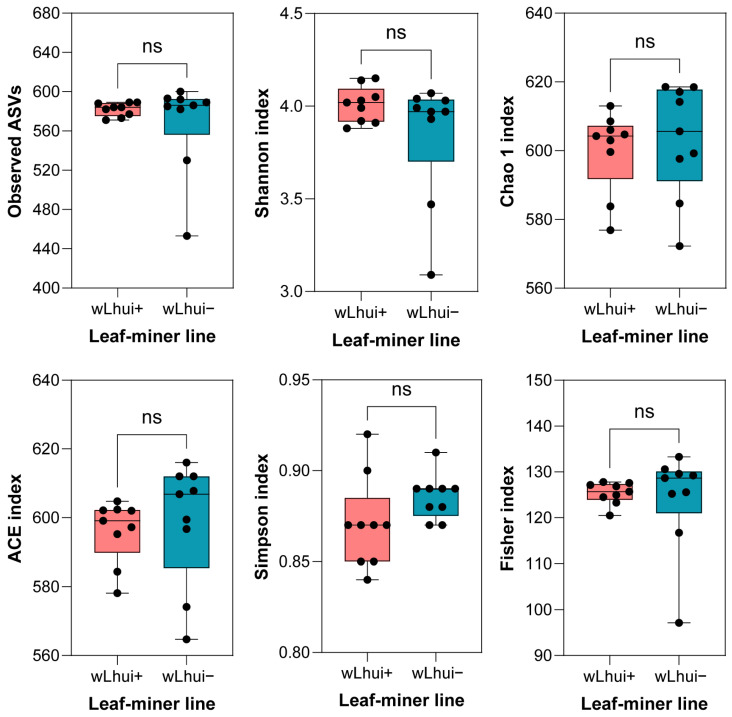
Bacterial alpha-diversity in *Wolbachia* wLhui-infected (wLhui+) and *Wolbachia*-uninfected (wLhui−) leaf-miners, as indicated by ASV observed richness, Shannon, Chao 1, ACE, Simpson, and Fisher indices. Boxplots represent the 10th and 90th percentiles (lower and upper boundaries), with the median indicated by the horizontal line inside each box. Statistical significance was assessed using the *Mann–Whitney U* test (ns, not significant).

The average number of observed ASVs in wLhui+ (n = 581.9) was slightly higher than in the wLhui− lines (n = 567.8) (Table S1). However, there were no significant differences in bacterial diversity between the two lines across all tested alpha-diversity indices (*Mann–Whitney U* test: ASV observed richness, *Z* = 30.50, *p* = 0.39; Shannon index, *Z* = 31.00, *p* = 0.42; Chao 1 index: *Z* = 33.00, *p* = 0.54; ACE index, *Z* = 28.00, *p* = 0.29; Simpson index, *Z* = 21.00, *p* = 0.07; Fisher index: *Z* = 28.00, *p* = 0.29) (Figure 1, and Table S1). These results suggest that *Wolbachia* infection does not significantly affect the alpha-diversity of the leaf-miner microbiome.

**Figure 2:**
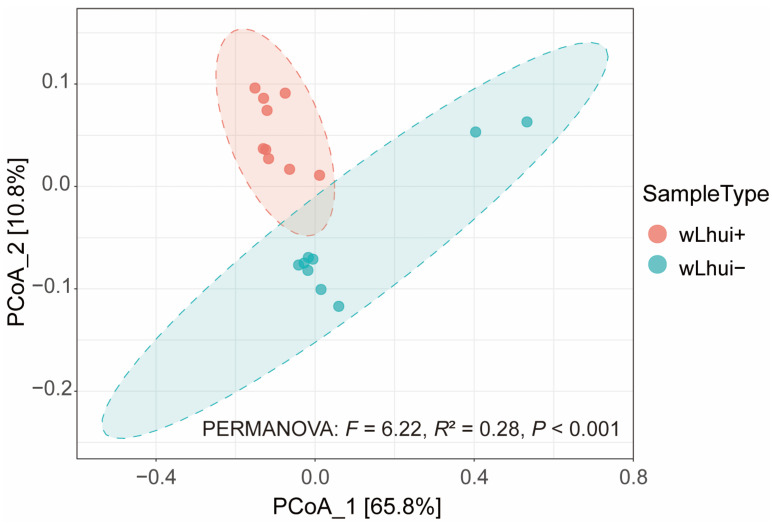
Bacterial beta-diversity of leaf-miners infected with or without *Wolbachia*. PCoA based on Bray–Curtis dissimilarities of *Wolbachia* wLhui-infected (wLhui+) and wLhui-uninfected (wLhui−) leaf-miners. The percentage of variance explained by the first two principal components is shown in parentheses. The significance of the differences in bacterial community composition was assessed using PERMANOVA (*p* < 0.05).

Principal coordinates analysis (PCoA) based on Bray–Curtis dissimilarities explained 76.7% of the variance in microbiota composition of the two leaf-miner lines, with PC1 accounting for 65.8% and PC2 accounting for 10.9% of the variance (Figure 2). PCoA revealed a distinct separation between the wLhui+ and wLhui− lines, suggesting a significant difference in microbial community structure between the two leaf-miner lines (PERMANOVA: *F* = 6.22, *R*^2^ = 0.28, *p* < 0.001) (Figure 2).

### 3.3. Impact of Wolbachia Infection on Microbiota Composition

**Figure 3:**
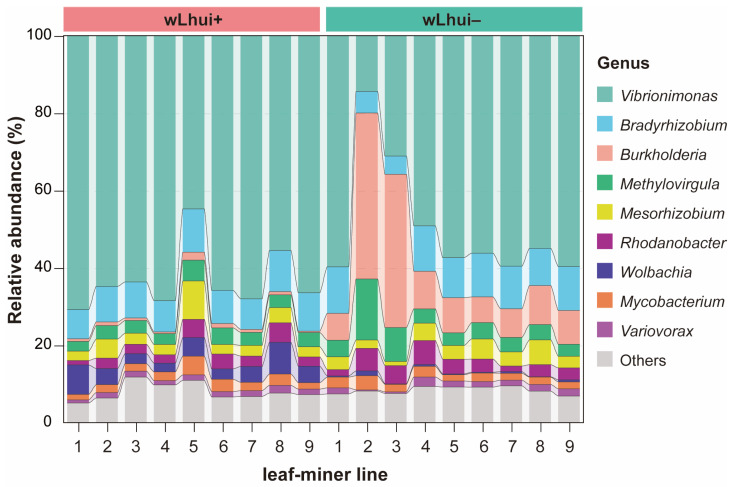
Relative abundance of bacterial genera in *Wolbachia* wLhui-infected (wLhui+) and -uninfected (wLhui−) leaf-miners. The relative abundance of the top bacterial genera is shown for each sample from both lines.

We next investigated the core microbial taxa within the wLhui+ and wLhui− lines. In the wLhui+ line, ten core genera (prevalence of >50%) accounted for more than 91% of the total abundance of the bacterial community (Figure S3). Dominant genera included *Vibrionimonas* (mean relative abundance of 63.52%), *Bradyrhizobium* (9.08%), *Wolbachia* (4.49%), *Mesorhizobium* (3.75%), *Methylovirgula* (3.53%), *Rhodanobacter* (2.97%), *Mycobacterium* (2.45%), and *Variovorax* (1.39%) (Figure 3; Figure S3). For the wLhui− line, eleven core genera were identified, constituting more than 94% of the total community composition (Figure 3; Figure S3). High-abundance genera in the wLhui− line included *Vibrionimonas* (50.2%), *Burkholderia* (14.83%), *Bradyrhizobium* (9.85%), *Methylovirgula* (5.37%), *Mesorhizobium* (3.7%), *Rhodanobacter* (3.59%), *Mycobacterium* (2.24%), *Ralstonia* (1.48%), and *Variovorax* (1.39%) (Figure 3; Figure S3). Seven core genera including *Bradyrhizobium*, *Mesorhizobium*, *Methylovirgula*, *Mycobacterium*, *Rhodanobacter*, *Vibrionimonas*, and *Variovorax* were shared between the two lines (Figure S3). However, heat tree analysis revealed significant differences in the relative abundance of some taxa between the two lines (Figure S4).

**Figure 4:**
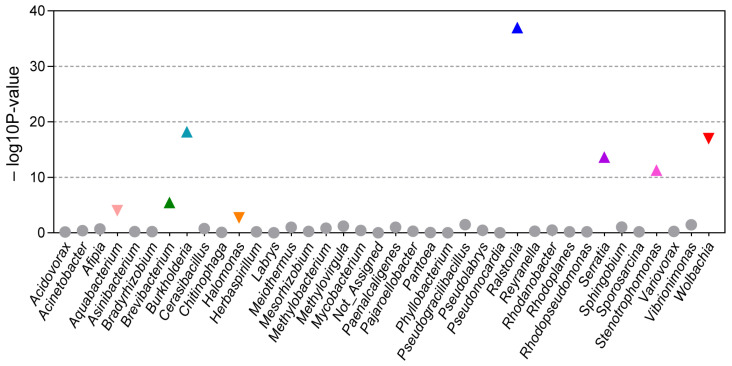
Variation in bacterial taxa between *Wolbachia* wLhui-infected (wLhui+) and -uninfected (wLhui−) leaf-miners. Significant differences in the relative abundance of bacterial genera between the two lines were calculated using DESeq2 analysis. Genera with log2 fold change (wLhui+ vs. wLhui−) > 2 and *p* < 0.05 were considered significantly different. The size of the points is determined by the logarithmic counts of genera between two lines. Upward-facing triangles indicate a positive fold change, while downward-facing triangles represent a negative fold change.

To further explore these differences, we used DESeq2 to identify bacterial taxa that exhibited significant changes in relative abundance between the wLhui+ and wLhui− lines. A total of eight genera displayed significant differences in relative abundance (Figure 4, Table S2). Notably, *Wolbachia* (*p* < 0.001, log2FC = 3.74), *Halomonas* (*p* < 0.001, log2FC = 1.51), and *Aquabacterium* (*p* < 0.001, log2FC = 3.09) were significantly more abundant in the wLhui+ line (Figure 4, Table S2). Conversely, *Ralstonia* (*p* < 0.001, log2FC = −4.53), *Stenotrophomonas* (*p* < 0.001, log2FC =−6.68), *Serratia* (*p* < 0.001, log2FC = −3.23), *Burkholderia* (*p* < 0.001, log2FC = −3.61), and *Brevibacterium* (*p* < 0.001, log2FC = −4.18) were significantly more abundant in wLhui− line (Figure 4, Table S2). LEfSe analysis confirmed these findings, showing higher abundance of *Wolbachia*, *Halomonas*, and *Aquabacterium* in the wLhui+ line compared to the wLhui− line (Figure S5).

### 3.4. Microbial Correlation Analysis

**Figure 5:**
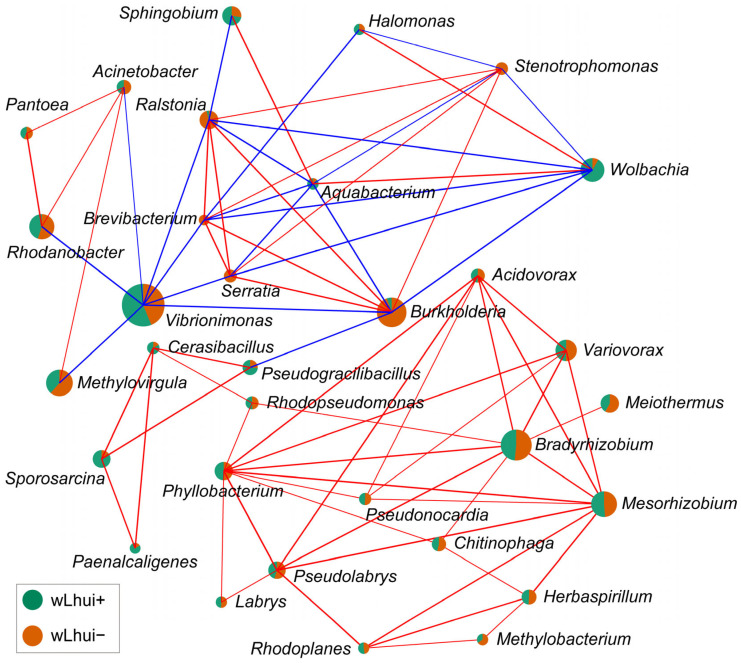
Correlation network of bacterial taxa (at the genus level) in both *Wolbachia* wLhui-infected (wLhui+) and wLhui-uninfected (wLhui−) leaf-miners. Pairwise microbe–microbe correlations were assessed using Spearman rank correlation, with a threshold of *p* < 0.05 and *|ρ|* > 0.6. The size of nodes represents the number of connections associated with each taxon. Green and orange shading within the nodes reflects the relative abundance of each genus in the wLhui+ or wLhui− lines, respectively. Positive correlations are represented by red edges, while negative correlations are indicated by blue edges. The thickness of the edges corresponds to the strength of the correlation. Statistical details are provided in Table S3.

Correlation network analysis revealed 77 significant microbial connections, including 56 positive and 21 negative correlations (*p* < 0.05, Spearman’s *ρ* > 0.6 or <–0.6) (Figure 5, Table S3). Specifically, in *Wolbachia*, the dominant endosymbiont in the wLhui+ line, showed a negative correlation with several genera, including *Serratia* (*ρ* = −0.729, *p* < 0.001), *Ralstonia* (*ρ* = −0.739, *p* < 0.001), *Stenotrophomonas* (*ρ* = −0.815, *p* < 0.001), *Burkholderia* (*ρ* = −0.709, *p* < 0.01), and *Brevibacterium* (*ρ* = −0.750, *p* < 0.001). On the other hand, *Wolbachia* exhibited strong positive correlations with *Halomonas* (*ρ* = 0.664, *p* < 0.01) and *Aquabacterium* (*ρ* = 0.758, *p* < 0.01) (Figure 5, Table S3).

## 4. Discussion

Uncovering the key drivers shaping microbial communities is pivotal for insect microbial ecology [4] as it offers valuable insights into the role of microbiota in the adaptation and spread of invasive species [49]. In this study, we observed a significant difference in the bacterial community structure between *Wolbachia* wLhui-infected and -uninfected leaf-miner lines. Notable shifts in the relative abundance of several dominant bacterial genera suggest that *Wolbachia* plays an important role in shaping the microbiota of leaf-miners, with potential yet-undetermined consequences for host fitness.

### 4.1. Core Microbe in the Leaf-Miner

We identified several core genera, including *Vibrionimonas*, *Bradyrhizobium*, *Mesorhizobium*, and *Methylovirgula*, which were highly abundant in both wLhui+ and wLhui− lines. These genera are commonly found across diverse environments such as insects, plants, water, and soil [58,59]. Our previous research, based on source tracking analysis, indicated that the leaf-miner likely acquires microbes from host plants or soil [52]. This stochastic acquisition may alter microbial composition [4,52,55]. However, vertically transmitted microbes like *Wolbachia* can exert priority effects, either facilitating or constraining changes in the microbiota [60]. Indeed, our findings here highlight complex interactions between *Wolbachia* and other microbes, evidenced by its positive and negative correlations with specific taxa. This underscores the importance of understanding how microbes are transmitted from the environment in unraveling the mechanisms driving bacterial community assembly in the leaf-miner.

### 4.2. Pervasive Effects of Wolbachia on the Host Microbiota

Our study revealed that the alpha-diversity of bacterial communities did not significantly differ between *Wolbachia* wLhui-infected and uninfected leaf-miner lines, aligning with previous studies in *Aedes aegypti* in which *Wolbachia* infection did not impact species diversity [61]. This may suggest that, despite the elimination of certain dominant microbes, such as *Wolbachia*, the bacterial diversity in the specific insect remains relatively stable. This finding may have important implications for maintaining host–microbe interactions, though further research is needed. In contrast, studies on other insect species, including *Delia radicum* [42], *Laodelphax striatellus* [37], *Drosophila melanogaster* [62], and *Sogatella furcifera* [38], have shown that *Wolbachia* infection significantly reduces microbial diversity. Given the differences among *Wolbachia* strains and their interactions with specific hosts, we speculated that the strength of *Wolbachia*’s effect on microbial diversity likely varies depending on the host species and *Wolbachia* strain, warranting further research across different taxa.

Despite the lack of significant changes in alpha-diversity, our results show that *Wolbachia* infection significantly modified bacterial community structure in *L. huidorbrensis*. This is consistent with controlled experiments and observational studies on other insects [37,38,40,41,61,62]. Notably, the extent to which *Wolbachia* alters the host microbiota varies across these systems, which may depend on the abundance of *Wolbachia* in the host and its ability to compete for nutrients with other bacterial species [61]. Additionally, some studies have reported negligible effects of *Wolbachia* on gut microbiota composition in *Drosophila melanogaster* [45] and *Anopheles stephensi* [44], suggesting that the impact of *Wolbachia* may be closely associated with host genotype and *Wolbachia* strain.

### 4.3. Underlying Mechanisms of Wolbachia’s Impact on the Host Microbiota

Three main hypotheses could explain how *Wolbachia* affects the microbiota in insects. First, coexisting microbes compete for limited resources and space within the host body, which could lead to the exclusion of less competitive microbes [21,22,30]. Our data suggest negative correlations between *Wolbachia* and genera such as *Serratia*, *Ralstonia*, and *Burkholderia*, indicating competitive interactions. Second, *Wolbachia* alters host metabolism and physiology, potentially suppressing the growth of specific bacterial taxa [37,63]. Changes in the intracellular environment, such as pH and reactive oxygen levels [40], might favor the survival of certain microbes. Our recent study demonstrated that *Wolbachia* wLhui modifies host cell metabolite profiles [56] and influences the expression of metabolism genes [37]. Third, some studies have suggested that *Wolbachia* may modulate host immune response [61,64], thus affecting microbial composition [65,66]. However, *Wolbachia* did not appear to alter immune responses in the small brown planthopper [37]. Currently, the precise mechanisms by which *Wolbachia* impacts the host microbiota remain unclear and warrant further investigation.

### 4.4. Limitations of the Study and Future Perspectives

It should be noted that antibiotics, commonly used to eliminate *Wolbachia* from insect systems, may also disrupt the host’s microbiome composition [67,68,69]. Although our antibiotic-treated leaf-miners were reared without antibiotics for over ten generations, allowing some microbes acquired from the environment to recover [52,55], which partially mitigates the impact of antibiotics on the host microbiota, the potential effects of antibiotics on certain microbes, especially those that cannot be acquired from the environment, cannot be entirely ruled out. To address this issue, further studies investigating the effect of *Wolbachia* on the host microbiota should focus on selecting *Wolbachia*-infected and uninfected leaf-miners from the same natural population. Additionally, 16S rRNA amplicon sequencing, the method we used for microbiome profiling, has limitations in distinguishing closely related species within genera and providing functional insights [69]. Future studies employing metagenomic or genomic approaches will address these limitations and provide a more comprehensive understanding of microbiome dynamics and functions of the specific microbe.

Moreover, the mutual influence of *Wolbachia* and microbiota and their combined impact on host phenotypes are often overlooked [70,71]. Our findings highlight the importance of considering microbiota composition when studying *Wolbachia*-induced phenotypic changes and in the design of microbe-based pest management strategies [5,6,72].

## 5. Conclusions

In summary, our study demonstrates that *Wolbachia* infection significantly alters the microbial community structure in *L. huidobrensis* but does not significantly affect microbial species diversity. We suggest that *Wolbachia* is the internal driver shaping the microbiota composition in leaf-miners, likely due to its interactions with other microbes. Future studies using metagenomic and genomic approaches are essential to elucidate the functional roles of *Wolbachia* and other microbes in the adaptation and invasiveness of the leaf-miner *L. huidobrensis*.

## Data Availability

All sequencing data are available at the Sequence Read Archive (SRA) under BioProject number PRJNA1187679.

## Supplementary Materials

The following supporting information can be downloaded at: https://www.mdpi.com/article/10.3390/microorganisms13020302/s1. Table S1: Alpha-diversity indices of microbiota in the *Wolbachia* wLhui-infected and -uninfected leaf-miner lines; Table S2: Statistical outputs of taxa abundance changes between wLhui+ and wLhui− lines; Table S3: Pairwise microbe–microbe correlation analysis in all samples; Figure S1. PCR detection of *Wolbachia* in the wLhui+ and wLhui− lines. M: DNA marker; ck+: positive control; ck-: negative control; Figure S2: Pie chart of the relative abundance of each phylum across all samples; Figure S3: Core genera taxa of the microbiome in the wLhui+ and wLhui− lines; Figure S4: Heat trees comparing the relative abundance of taxa between wLhui+ and wLhui− lines. Taxa colored in green and red are enriched in wLhui− and wLhui+ lines, respectively; Figure S5: The linear discriminant analysis effect size (LEfSe) analysis of differential genus abundance in the wLhui+ line compared to the wLhui− line.
