# Supplementary material for: Wolbachia Infection Alters the Microbiota of the Invasive Leaf-Miner Liriomyza huidobrensis (Diptera: Agromyzidae)
Source: Microorganisms. 2025 Jan 30;13(2):302. doi: 10.3390/microorganisms13020302 (PMC11858490; doi:10.3390/microorganisms13020302)
Supplement: Supplementary file 1 [file microorganisms-13-00302-s001.zip › microorganisms-3406586-supplementary.pdf]

microorganisms

MDPI

Supplementary Materials

Table S1. Alpha-diversity indices of microbiota in the *Wolbachia* wLhui-infected and -uninfected leaf-miner lines

Samples	Observed ASVs	Shannon	Chao 1	ACE	Simpson	Fisher	Goods coverage (%)
wLhui+_1	582	3.92	603.00	595.23	0.84	124.48	99.68
wLhui+_2	588	4.03	604.25	602.15	0.87	126.85	99.68
wLhui+_3	584	4.05	606.02	599.10	0.87	125.67	99.65
wLhui+_4	573	4.15	583.78	584.36	0.87	123.26	99.74
wLhui+_5	577	3.99	604.73	602.00	0.92	127.77	99.44
wLhui+_6	589	4.14	608.52	602.37	0.87	127.12	99.68
wLhui+_7	589	3.91	613.00	604.76	0.85	124.93	99.65
wLhui+_8	584	4.02	599.65	597.23	0.90	127.55	99.66
wLhui+_9	571	3.88	576.88	578.16	0.85	120.51	99.82
wLhui–_1	592	4.07	617.14	607.78	0.89	128.65	99.63
wLhui–_2	453	3.09	572.23	564.70	0.87	97.14	98.56
wLhui–_3	530	3.47	584.64	574.13	0.88	116.70	99.13
wLhui–_4	600	4.04	618.56	616.04	0.91	133.25	99.58
wLhui–_5	585	3.97	605.63	606.83	0.89	129.21	99.53
wLhui–_6	589	4.03	618.50	612.05	0.89	130.58	99.49
wLhui–_7	586	3.93	597.64	599.46	0.87	125.55	99.70
wLhui–_8	593	3.99	614.20	612.01	0.89	129.59	99.57
wLhui–_9	582	3.97	599.20	596.71	0.88	125.20	99.66

Table S2. Statistical outputs of taxa abundance changes between wLhui+ and wLhui- lines

Taxa	log2FC	lfcSE	Pvalues	FDR
Acidovorax	0.10091	0.27806	0.71669	0.85107
Acinetobacter	-0.80149	0.93334	0.39049	0.70659
Afipia	0.39749	0.30586	0.19374	0.43307
Aquabacterium	3.0952	0.79485	9.86E-05	0.00053516
Asinibacterium	0.18251	0.3368	0.58789	0.82517
Bradyrhizobium	0.071296	0.13787	0.60506	0.82517
Brevibacterium	-4.1829	0.90122	3.46E-06	2.19E-05
Burkholderia	-3.6188	0.4071	6.14E-19	1.17E-17
Cerasibacillus	1.4771	1.0926	0.17643	0.41903
Chitinophaga	-0.064879	0.30746	0.83287	0.95907
Halomonas	1.5161	0.49033	0.0019875	0.0094407
Herbaspirillum	0.11955	0.25088	0.63372	0.82517
Labrys	0.041947	0.47946	0.93028	0.96338
Meiothermus	-0.3026	0.18297	0.098173	0.27089
Mesorhizobium	0.14923	0.24004	0.53415	0.82517
Methylobacterium	-0.74163	0.509	0.14511	0.36761
Methylovirgula	-0.68922	0.36868	0.061566	0.21268
Mycobacterium	0.1856	0.20765	0.37142	0.7057
Not_Assigned	-0.0034416	0.28114	0.99023	0.99023
Paenalcaligenes	2.406	1.4619	0.099802	0.27089
Pajaroellobacter	-0.22971	0.35978	0.52317	0.82517
Pantoea	-0.18289	1.1198	0.87026	0.96338
Phyllobacterium	0.029472	0.32023	0.92667	0.96338
Pseudogracilibacillus	1.7248	0.80384	0.031896	0.13395
Pseudolabrys	-0.17332	0.17999	0.33558	0.67115
Pseudonocardia	0.032712	0.42072	0.93802	0.96338
Ralstonia	-4.5326	0.35318	1.07E-37	4.05E-36
Reyranella	0.23865	0.3791	0.52901	0.82517
Rhodanobacter	-0.31349	0.3179	0.32407	0.67115
Rhodoplanes	0.31913	0.72925	0.66167	0.82517
Rhodopseudomonas	0.12527	0.29698	0.67317	0.82517
Serratia	-3.232	0.42339	2.28E-14	2.17E-13
Sphingobium	1.6068	0.94846	0.090241	0.27089
Sporosarcina	-0.21438	0.47487	0.65167	0.82517
Stenotrophomonas	-6.6887	0.96914	5.14E-12	3.91E-11
Variovorax	0.16287	0.26847	0.54408	0.82517
Vibrionimonas	0.57182	0.27159	0.035249	0.13395
Wolbachia	3.7428	0.43691	1.07E-17	1.35E-16

Table S3. Pairwise microbe-microbe correlation analysis in all samples

Taxon1	Taxon2	Correlation	P.value	Statistic
Acidovorax	*Bradyrhizobium*	0.6367	0.0045	352.00
Acidovorax	*Mesorhizobium*	0.7998	1.00E-04	194.00
Acidovorax	*Phyllobacterium*	0.7544	3.00E-04	238.00
Acidovorax	*Pseudolabrys*	0.7152	8.00E-04	276.00
Acidovorax	*Pseudonocardia*	0.6512	0.0034	338.00
Acidovorax	*Variovorax*	0.6966	0.0013	294.00
Acinetobacter	*Methylovirgula*	0.7688	2.00E-04	224.00
Acinetobacter	*Not_Assigned*	0.6718	0.0023	318.00
Acinetobacter	*Pantoea*	0.6822	0.0018	307.95
Acinetobacter	*Rhodanobacter*	0.709	0.001	282.00
Acinetobacter	*Vibrionimonas*	-0.6883	0.0016	1636.00
Aquabacterium	*Brevibacterium*	-0.6758	0.0021	1623.86
Aquabacterium	*Burkholderia*	-0.7517	3.00E-04	1697.36
Aquabacterium	*Ralstonia*	-0.7984	1.00E-04	1742.69
Aquabacterium	*Serratia*	-0.6755	0.0021	1623.52
Aquabacterium	*Sphingobium*	0.7878	1.00E-04	205.61
Aquabacterium	*Stenotrophomonas*	-0.8199	0	1763.48
Aquabacterium	*Wolbachia*	0.758	3.00E-04	234.46
Bradyrhizobium	*Chitinophaga*	0.7131	9.00E-04	278.00
Bradyrhizobium	*Meiothermus*	0.6904	0.0015	300.00
Bradyrhizobium	*Mesorhizobium*	0.6821	0.0018	308.00
Bradyrhizobium	*Phyllobacterium*	0.8287	0	166.00
Bradyrhizobium	*Pseudolabrys*	0.7028	0.0011	288.00
Bradyrhizobium	*Rhodopseudomonas*	0.6739	0.0022	316.00
Bradyrhizobium	*Variovorax*	0.7296	6.00E-04	262.00
Brevibacterium	*Burkholderia*	0.8258	0	168.82
Brevibacterium	*Halomonas*	-0.6619	0.0028	1610.33
Brevibacterium	*Ralstonia*	0.764	2.00E-04	228.64
Brevibacterium	*Serratia*	0.8041	1.00E-04	189.78
Brevibacterium	*Stenotrophomonas*	0.8433	0	151.82
Brevibacterium	*Vibrionimonas*	-0.6273	0.0053	1576.88
Brevibacterium	*Wolbachia*	-0.7508	3.00E-04	1696.54
Burkholderia	*Pseudogracilibacillus*	-0.6244	0.0056	1574.00
Burkholderia	*Ralstonia*	0.8679	0	128.00
Burkholderia	*Serratia*	0.731	6.00E-04	260.63
Burkholderia	*Stenotrophomonas*	0.8099	0	184.21
Burkholderia	*Vibrionimonas*	-0.8019	1.00E-04	1746.00
Burkholderia	*Wolbachia*	-0.709	0.001	1656.00
Cerasibacillus	*Paenalcaligenes*	0.7532	3.00E-04	239.17
Cerasibacillus	*Pseudogracilibacillus*	0.6142	0.0067	373.83
Cerasibacillus	*Rhodopseudomonas*	0.633	0.0048	355.64
Cerasibacillus	*Sporosarcina*	0.7706	2.00E-04	222.25
Chitinophaga	*Herbaspirillum*	0.6821	0.0018	308.00
Chitinophaga	*Phyllobacterium*	0.645	0.0038	344.00
Halomonas	*Stenotrophomonas*	-0.6569	0.0031	1605.53

Halomonas	*Wolbachia*	0.6649	0.0026	324.67
Herbaspirillum	*Mesorhizobium*	0.7152	8.00E-04	276.00
Herbaspirillum	*Methylobacterium*	0.6144	0.0067	373.69
Herbaspirillum	*Rhodoplanes*	0.6256	0.0055	362.75
Labrys	*Phyllobacterium*	0.6182	0.0063	370.00
Labrys	*Pseudolabrys*	0.6553	0.0032	334.00
Mesorhizobium	*Phyllobacterium*	0.9071	0	90.00
Mesorhizobium	*Pseudolabrys*	0.9174	0	80.00
Mesorhizobium	*Pseudonocardia*	0.6904	0.0015	300.00
Mesorhizobium	*Rhodoplanes*	0.7001	0.0012	290.60
Mesorhizobium	*Variovorax*	0.6285	0.0052	360.00
Methylobacterium	*Rhodoplanes*	0.6829	0.0018	307.29
Methylovirgula	*Vibrionimonas*	-0.6326	0.0048	1582.00
Paenalcaligenes	*Sporosarcina*	0.6773	0.002	312.71
Pantoea	*Rhodanobacter*	0.7106	9.00E-04	280.45
Phyllobacterium	*Pseudolabrys*	0.9009	0	96.00
Phyllobacterium	*Pseudonocardia*	0.612	0.007	376.00
Phyllobacterium	*Rhodopseudomonas*	0.6553	0.0032	334.00
Phyllobacterium	*Variovorax*	0.6594	0.0029	330.00
Pseudogracilibacillus	*Sporosarcina*	0.7771	1.00E-04	216.00
Pseudolabrys	*Rhodoplanes*	0.7663	2.00E-04	226.47
Pseudonocardia	*Variovorax*	0.7214	7.00E-04	270.00
Ralstonia	*Serratia*	0.7269	6.00E-04	264.64
Ralstonia	*Sphingobium*	-0.6223	0.0058	1572.00
Ralstonia	*Stenotrophomonas*	0.8606	0	135.03
Ralstonia	*Vibrionimonas*	-0.6099	0.0072	1560.00
Ralstonia	*Wolbachia*	-0.7399	4.00E-04	1686.00
Rhodanobacter	*Vibrionimonas*	-0.7626	2.00E-04	1708.00
Serratia	*Stenotrophomonas*	0.7822	1.00E-04	211.03
Serratia	*Vibrionimonas*	-0.6371	0.0045	1586.32
Serratia	*Wolbachia*	-0.729	6.00E-04	1675.36
Stenotrophomonas	*Wolbachia*	-0.8153	0	1759.02

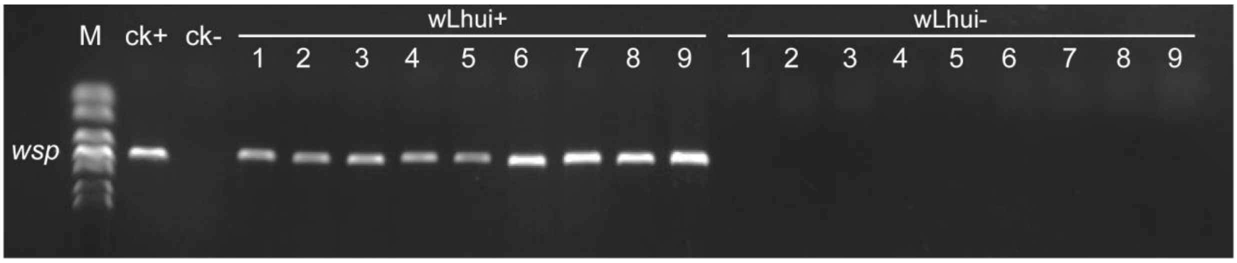

Figure S1. PCR detection of *Wolbachia* in the wLhui+ and wLhui- lines. M: DNA marker; ck+: positive control; ck-: negative control.

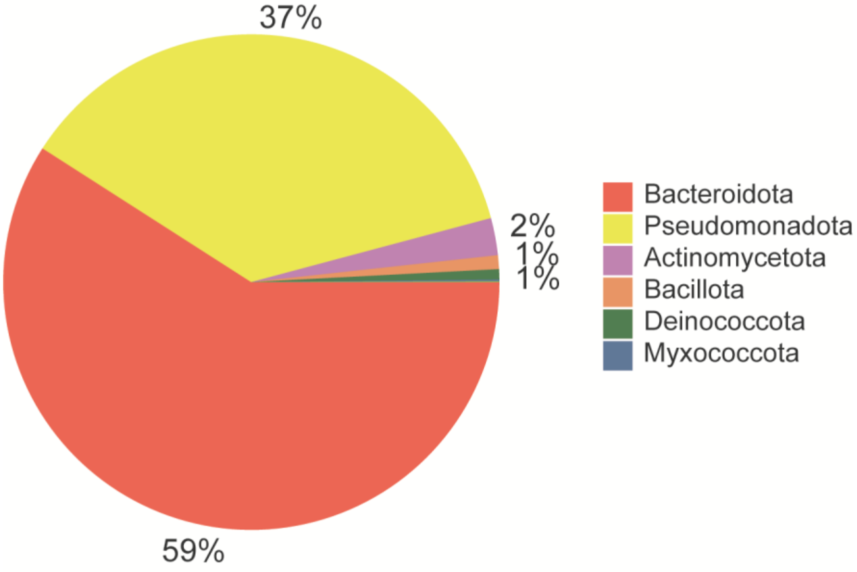

Figure S2. Pie chart of the relative abundance of each phylum across all samples

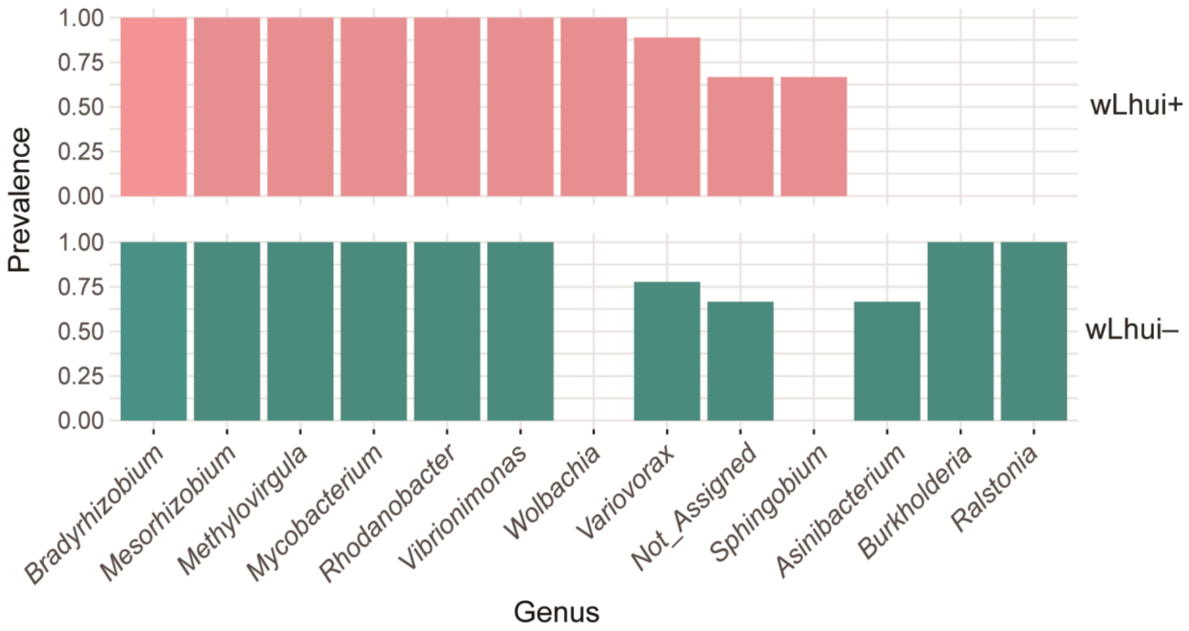

Figure S3. Core genera taxa of the microbiome in the wLhui+ and wLhui- lines

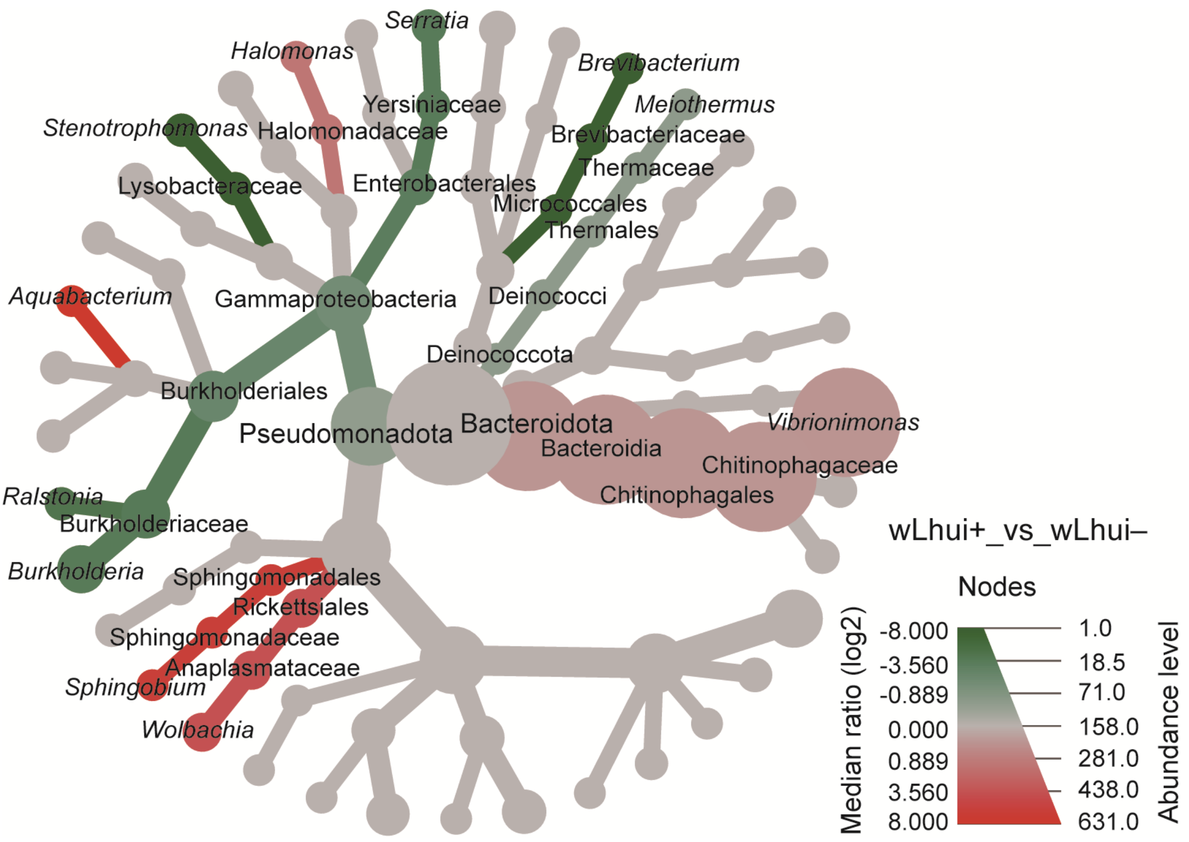

Figure S4. Heat trees comparing the relative abundance of taxa between wLhui+ and wLhui- lines. Taxa colored in green and red are enriched in wLhui- and wLhui+ lines, respectively

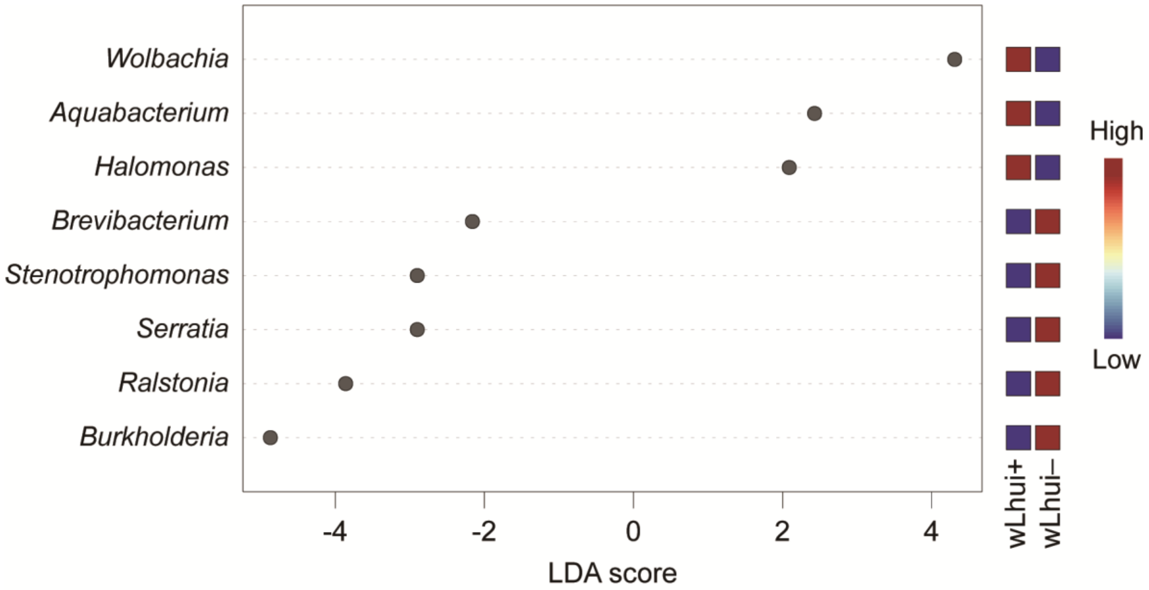

Figure S5. The linear discriminant analysis effect size (LEfSe) analysis of differential genus abundance in the wLhui+ line compared to the wLhui- line
